# Case vignettes for simulated patients based on real patient experiences in the context of OSCE examinations: workshop experiences from interprofessional education

**DOI:** 10.3205/zma001487

**Published:** 2021-06-15

**Authors:** Andrea Glässel, Peter Zumstein, Theresa Scherer, Emanuel Feusi, Nikola Biller-Andorno

**Affiliations:** 1 Zurich University of Applied Sciences (ZUAS), Interprofessional Education and Collaborative Practice Unit (IECP), Institute of Health Sciences (IHS), Winterthur, Switzerland; 2 University of Zurich, Institute of Biomedical Ethics and History of Medicine (IBME), Zurich, Switzerland; 3 University of Applied Sciences (UAS) Bern, Department of Health Bern, Competence Centre for Interprofessional Education, Bern, Switzerland

**Keywords:** patient-centredness, simulated patients, OSCE, real patient narratives, interprofessional education, healthcare professions

## Abstract

**Background:** Patient-centredness (PCN) is an increasingly demanded objective in health care and has gained importance for the care situation, for research, and the education of healthcare professions. The literature shows that the term PCN is not uniformly defined. Key aspects for the concept of PCN can be found in the integrative model and its dimensions by Scholl and colleagues (2014), which are incorporated into the acquisition of competencies in Objective Structured Clinical Examination (OSCE) examination formats. The inclusion of subjective experiences of persons directly affected in health-related situations is recognized as an important factor for continuous improvement in health care. In the interprofessional education of healthcare professions, subjective experiences serve as a starting point in relation to OSCE exams. In this context, the project “DIPEx” “Database of Individual Patients’ Experiences” stands for the systematic collection and evaluation of subjective experiences of illness using scientific methods.

**Aim: **The aim of this interprofessional training workshop was to show

1. how PCN can support the writing of case vignettes based on real experiences from systematically collected narratives within the DIPEx project, as well as
2. the preparation of simulation subjects for OSCE examinations in the healthcare professions.

**Methods: **Interactive, moderated workshop with two theory-based input presentations on the systematic development of interprofessional case vignettes based on four steps, group work with synthesis in the form of statements, and a concluding outlook.

**Results: **With regard to the aims of the workshop, the synthesis included results at two levels:

1. exemplary results on aims not explicitly addressed: Interprofessional teaching is full of presuppositions and requires clarification of four different perspectives in advance to be included in case development;
2. exemplary results on explicitly addressed aims: Listening to and incorporating the real-life experiences and narratives of persons directly affected in health-related situations and their families was seen as an important learning aspect for PCN in relation to the practitioner-patient relationship.

Five key statements on explicit aims for case development involving PCN emerged from the group work.

**Conclusion: **Competency-based interprofessional education of health professionals and PCN can benefit from real patient narratives of health and illness as simulated patients can portray roles in OSCE formats in a realistic and convincing manner.

## Zusammenfassung

**Hintergrund: **Patient*innenorientierung (PO) ist in der Gesundheitsversorgung ein zunehmend gefordertes Ziel und hat für die Versorgungssituation, Forschungslage und für die Ausbildung von Gesundheitsberufen an Bedeutung gewonnen. In der Literatur zeigt sich, dass der Begriff PO nicht einheitlich definiert ist. Zentrale Aspekte für das Konzept der PO finden sich im integrativen Modell und seinen Dimensionen von Scholl und Kollegen aus 2014, die in den Kompetenzerwerb bei Objective Structured Clinical Examination (OSCE) Prüfungsformaten Eingang finden. Der Einbezug subjektiver Erfahrungen von Betroffenen wird als wichtiger Faktor für die kontinuierliche Verbesserung der Gesundheitsversorgung anerkannt. In der interprofessionellen Ausbildung der Gesundheitsberufe dienen sie als Ausgangspunkt in Bezug auf OSCE Prüfungen. Das Projekt „DIPEx“ „Database of Individual Patients’ Experiences“ steht dabei für die systematische Erhebung und Auswertung subjektiver Krankheitserfahrungen anhand wissenschaftlicher Methoden.

**Ziel des Beitrags:** Ziel des Interprofessionellen Ausbildungs-Workshops war es aufzuzeigen, wie

1. PO anhand realer Erfahrungen aus systematisch erhobenen Narrativen im Rahmen des DIPEx-Projektes das Verfassen von Fallvignetten unterstützen kann, wie auch
2. die Vorbereitung von Simulationspersonen für OSCE Prüfungen in den Gesundheitsberufen.

**Methode:** Interaktiver, moderierter Workshop mit zwei theoriegeleiteten Inputpräsentationen zur systematischen Entwicklung interprofessioneller Fallvignetten anhand von vier Schritten in Gruppenarbeit mit Synthesebildung in Form von Statements und abschliessendem Ausblick.

**Ergebnisse: **Mit Bezug auf die Ziele des Workshops umfasste die Synthese Ergebnisse auf zwei Ebenen:

1. exemplarische Ergebnisse zu nicht explizit adressierten Zielen: Interprofessionelle Lehre ist voraussetzungsreich und verlangt vorab die Klärung von vier verschiedenen Perspektiven, die in die Fallentwicklung einbezogen werden;
2. exemplarische Ergebnisse zu explizit adressierten Zielen: Das Zuhören und der Einbezug der realen Erfahrungen und Erzählungen von Betroffenen und ihren Angehörigen wurde als wichtiger Lernaspekt für PO in Bezug auf die Behandler*innen-Patienten-Beziehung gesehen.

Aus der Gruppenarbeit gingen fünf zentrale Statements zu expliziten Zielen für die Fallentwicklung unter Einbezug der PO hervor.

**Fazit:** Die kompetenzorientierte interprofessionelle Ausbildung der Gesundheitsberufe und PO kann von realen Patient*innennarrativen zu Gesundheit und Krankheit profitieren, indem Simulationspersonen in OSCE-Formaten Rollen realitätsnah und überzeugend darstellen können.

## 1. Background patient-centredness

**Figure 1:**
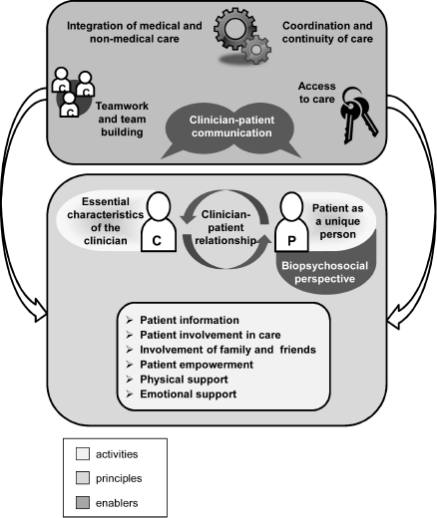
The integrative Model of Patient-Centredness [5]

Patient-centredness (PCN) is an increasingly demanded aim in health care [1], [2]. Moreover, in recent years the topic has gained importance for the health care situation, for research, and for the education of healthcare professions. However, the literature shows that conceptually the term patient-centredness is not uniformly defined [3], [4], because models and definitions include different dimensions [5]. On the one hand, PCN points to a socially demanded new image of the patient, placing empowered and informed patients alongside medical experts who advocate strengthening patient autonomy [6]. On the other hand, the term PCN includes the orientation of structures, processes, and outcomes of the health care system, as well as the respect of interests, needs and wishes of individual patients. Patients experience empathy, receive beneficial and desirable services, and are granted their rights and responsibilities [7]. Central aspects, such as the dimensions physical and psychological support, patient information, empowerment, a biopsychosocial perspective, the practitioner-patient communication, as well as the practitioner-patient relationship and others are bundled in the integrated model for PCN by Scholl and colleagues (2014) [5] (see figure 1 (Fig. 1)). 

## 2. Competence-oriented education of healthcare professions including PCN with simulated patients in preparation for “Objective Structured Clinical Examination” formats

The model of Scholl and colleagues (2014) [5], used as a theoretical-conceptual framework of PCN, is more and more integrated in the acquisition of competences. Thus, PCN is an examination subject for students of healthcare professions. Competences are a key reference value in vocational training for healthcare professions, to which examinations and their formats should be aligned. The term “competence” describes “the ability to apply knowledge and skills acquired through experience and learning independently, responsibly and appropriately in always new situations” ([8], p.148). At the latest with the Lancet report by Frenk et al [9] published in 2010, competence orientation moved in into universities for health professionals, nursing and medicine and has since been strived for to be implemented by means of various didactic approaches. To meet the demand of competence orientation, the examination format “Objective Structured Clinical Examination (OSCE)” is focused on, a format that has found a high degree of acceptance in the teaching of healthcare professions [10].

The focus is on the objective, structured assessment of clinical competence, as well as decision-making competence with reference to aspects of action. For a high standardization of action processes, within the framework of OSCE simulated persons (SP) are engaged, who form an integral part of today’s teaching of health professionals [9]. SP refers to actors and actresses trained to perform patient roles within teaching contexts to facilitate credible practice, examination, and feedback scenarios at a high level of standardization. In the context of competence acquisition, SPs are thus required to include conceptual bases of PCN also in OSCE examinations and, to this purpose, to relate to frameworks such as the dimensions of the integrated model of Scholl and colleagues (2014) [5]. This could mean, for instance, to design the role descriptions for the following aspects:

1. to convincingly simulate realistic situations regarding good or bad practitioner-patient communication;
2. to integrate a biopsychosocial perspective of functioning for practicing patient-oriented goal formulations; or
3. to include aspects reflecting the practitioner-patient relationship, and also for further aspects.

To fulfil their role, SPs should be able to take the perspective of the persons affected in health-related situations and to know multiple symptoms with their life-changing consequences for these persons [5]. Gaining insight into subjective experiences from the patients’ point of view and into related stories of their life and disease can support the SP in this process. Individual, real experiences from the perspective of the person affected are gaining increasing importance [11]. In addition, listening to and including the experiences of individuals directly affected and their families is recognized as an important factor for continuous improvement of health care. Experiences are mostly described in the form of narratives and are accessible, for example, via the Internet, as described in the following project “DIPEx”.

## 3. Project DIPEx – Database for real patient experiences based on narrative interviews

**Figure 2:**
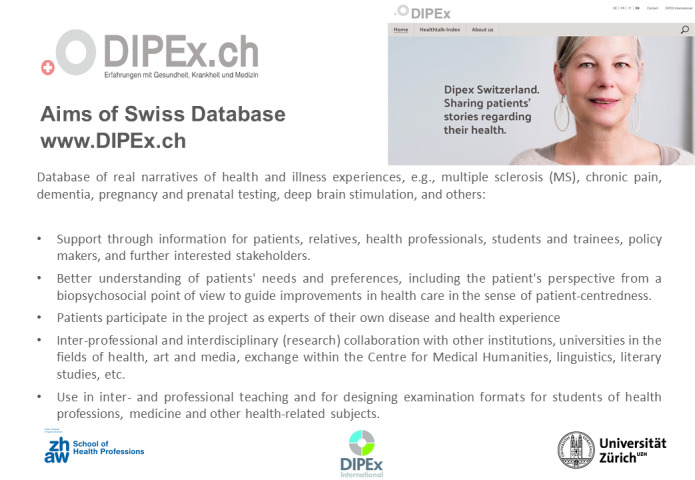
Goals of the Swiss database http://www.dipex.ch from 2021

The project “DIPEx” stands for “**D**atabase of **I**ndividual **P**atients' **Ex**periences”, originated in England in 2000, in which real patient experiences are collected, are processed by the Health Experiences Research Group (HERG) of the University of Oxford, and are made available on the Internet at [https://healthtalk.org/]. The DIPEx project is embedded into an international research network [https://dipexinternational.org/], which now includes 14 countries, among them Switzerland, Germany, the USA, Japan, the Netherlands, and others. The DIPEx websites cover more than 100 different diseases or health topics. The special feature of the project is the systematic collection, evaluation, and presentation of subjective experiences of health and disease based on established scientific methods that are defined, manualized and reviewed within the research network [https://www.krankheitserfahrungen.de/], [12]. Thus, the project differs from the currently diverse forms of using patient narratives in social media such as forums, blogs, or commercial sites on the Internet [13]. The overall aim of the project (see figure 2 (Fig. 2)) is to meet the diverse information needs of patients and their caregivers, but also to familiarize all stakeholders, including students in health and social care professions, with the perspective of patients and to stimulate PCN in its various dimensions [14], [15].

## 4. Aim of the report

This paper outlines excerpts from a workshop held at a conference on Interprofessional Education (IPE) at the Bern University of Applied Sciences (BUAS), in cooperation with the Zurich University of Applied Sciences (ZUAS) Winterthur in February 2020. The aim of this workshop was to show

1. how PCN can support the writing of case vignettes^1^, as well as
2. the preparation of simulation subjects for "OSCE" exams in the healthcare professions, based on real patient experiences from systematically collected narratives from the DIPEx project.

## 5. Method

The 90-minute IPE workshop entitled: *“Learning situations with case vignettes: instruction to work with cases that is conducive to interprofessional engagement”*, consisted of the following six steps and was led by two co-facilitators (AG&PZ):

1. Welcome, explanations on how to proceed, recall of participants’ experiences on case development.
2. Input 1: Theoretical introduction to case development for SPs and conceptual framing of PCN.
3. Input 2: Presentation of DIPEx project including the theoretical concept of PCN.
4. Work in small groups on statements of IPE as basis for case development.
5. Presentation of results and synthesis.
6. Outlook and conclusion.

### Input 1: Introduction to case development for SPs and conceptual framing to PCN 

The 15-minute input presentation introduced the systematic case development for OSCE with SPs based on four steps. In Miller’s learning pyramid [16] OSCE tests students at the level of “shows how”, using different stations in a structured way. Students should demonstrate their competencies to be tested involving PCN [17]. Therefore, the starting point was the development of an interprofessional case vignette with reference to selective dimensions of PCN from the integrative model of Scholl et al 2014 [5]. In order to design the roles of SPs short examples were developed,, first orally in the plenary and then in writing in the small groups, e.g. the inclusion of a biopsychosocial perspective for the formulation of treatment goals, or aspects that are conducive to or might hinder practitioner-patient communication (see chapters 1 and 2) . 

#### Input 2: Presentation of the DIPEx project including the theoretical concept of the PCN

As introduced in chapter 3, the 15-minute input presentation first outlined core contents of the project including video examples from DIPEx Germany, as well as the interactive exchange on two studies from teaching with DIPEx [18], [19]. Obtaining subjective experiences from the patient's perspective [20], [21], via text, video or audio contribution served to contextualize the case events for the participants. Real patient narratives thereby show the connection to the dimensions of PCN from the model of Scholl et al. (2014) [5] and help to prepare for the role of SP for OSCE. The short examples from input 1 mentioned above were related to the video examples shown. This was followed by the introduction to the group work assignment.

#### Interactive group work with presentation of results and synthesis

Two small groups of five people each were formed including the two co-facilitators. The task was to discuss the content from input 1 and 2 and to reflect on guiding questions for an interprofessional case vignette. From the reflection, statements for the case development were recorded on flip charts. The moderated presentation of the results was followed by a joint synthesis of the results of the two groups. From this, the steps for the next IP workshop on case development in the fall of 2020 were derived.

## 6. Results

The results of the IP workshop show that the eight participants (five female) from four health professions (physiotherapy, occupational therapy, midwifery, and nursing) had diverse experience with interprofessional case development. However, they were breaking new ground in terms of a structured approach for case development for OSCE examinations and in working with SPs, except for one participant. 

The synthesis of the workshop includes outcomes at the following two levels: 

Level 1 in terms of outcomes that did not explicitly address the aims of the workshop.

Level 2 in terms of outcomes that explicitly addressed the two aims of the workshop:

- Aim 1: To support PCN using real patient experiences from systematically collected narratives.
- Aim 2: To support the preparation of simulation subjects for "OSCE" examinations in the healthcare professions.

Level 1: Outcomes that did not explicitly address the aims of the workshop: this related to setting specific prerequisites for the workshop and about participants who had no prior experience in working collaboratively on case vignettes for OSCE.

1. Interprofessional teaching is full of presuppositions and requires clarification in advance of perspectives that will be included in case development. The following four perspectives were discussed: the student perspective, the faculty perspective, the patient perspective, and the simulation subject perspective.
2. Before the development of a new case vignette can be actively started, terms and action steps for case development need to be clarified together, such as a common interprofessional understanding of PCN, examination content, aims and competencies. The theoretical-conceptual framework on PCN was useful to address these issues and served as a starting point.

Level 2: Outcomes that explicitly addressed the aims of the workshop:

1. Listening to and including real experiences and narratives of patients and their relatives was seen as an important learning aspect for PCN in relation to the practitioner-patient relationship. Furthermore, this is recognized as a significant factor for the continuous improvement of health care.
2. It was important to be informed about systematic and non-systematic narratives and to receive knowledge of where systematically collected narratives are available.
3. Video contributions to real narratives provide a first impression of the person and the situation in a simple way. Even in short film sequences, non-verbal signs such as the gaze, gestures or body posture are help simulated patients to shape their role more specifically at a later stage.
4. Five central statements emerged from the group work on explicit aims for case development involving PCN. These are summarized in table 1 (Tab. 1).

## 7. Discussion

On the one hand, the IP workshop served as a basis for writing interprofessional oriented case vignettes for the healthcare professions incorporating theoretical concepts on PCN. On the other hand, it was intended to support SPs in preparing competency-based examination formats, such as for OSCE examinations. Real patient experiences from systematically collected narratives were proposed for writing the case vignettes. Patient narratives serve as so-called “role models” so that SPs can convey practical relevance, convincingly assume the facets of their roles, and present the patient perspective. The intention is to integrate real patient experiences into the role of the case vignette in a lifelike manner. The true-to-life portrayal gives the SP a higher degree of credibility, persuasiveness, and identification with the role. Video and audio contributions from the DIPEx websites [https://healthtalk.org/], [https://www.krankheitserfahrungen.de/] and in the future [https://dipex.ch/] provide a variety of real-life templates for this purpose in order to embed them directly into the script of the vignette to the minute (see table 1 (Tab. 1)). *“As a writer (...) of a role, writing a role becomes easier if you are inspired by a person from your working or living environment (...).”* ([10], p.37). 

Initial results from an RCT study were able to show that medical students benefit from real patient narratives and performed more competently in terms of their communication skills than students in the comparison group who were taught content by subject matter experts [18]. Results from interprofessional learning settings (physical therapy and social work) support this initial evidence [19]. Narrative interviews represent a possible access to experiences in this context. They open the perspective on PCN regarding the inner experience of illness, experiences with the health care system, treatment successes or failures, and coping with illness [22]. With regard to strengthening PCN, Charon 2007 and Shao-Yin et al. 2020 could show that the ability of active listening is to be considered as a central influencing variable for the practitioner-patient relationship, which is associated with appreciation, respect, and empathy, among others [23], [24]. Video, audio or text contributions of systematically collected online patient narratives can support teachers and SPs in strengthening this skill in students in order to develop it as a sub-component for narrative competence and to test it accordingly, for example with OSCE formats [24], [25]. 

Among the limitations of the workshop, it should be noted that this was a first introduction to the topic. Thus, the different theoretical concepts on PCN, or also on the methodological procedure within the writing process for a case vignette, were not introduced in depth. The composition of the interprofessional group of participants was random. A limited time window was available to discuss content and questions for the workshop assignment and to write up the results in the form of statements. If running the workshop again, it was suggested that the time window for the group work be extended by at least 45 minutes. Follow-up tasks and wishes for a follow-up workshop were noted. 

## 8. Conclusion

This IP workshop laid the foundation for developing an initial common understanding for the development of future case vignettes for OSCE examinations. The theoretical concept on PCN served as a common starting point. A continuation of this workshop is already in preparation and will follow in the form of an “online atelier” as a writing workshop for case development. For patient-centredness the competence-oriented interprofessional education in the healthcare professions (including physicians) can benefit from the embedding of real patient narratives on health and illness as simulated patients in OSCE formats can portray patient roles convincingly and more realistically.

## Note

^1^ The instructions for writing up the case vignettes are described in a separate article.

## Acknowledgements

We would like to thank the highly motivated participants of the workshop, who discussed the guiding questions on case development in a lively and constructive way and openly shared and presented their reflections on them. Furthermore, we would like to thank Brigitta Spiegel- Steinmann (Interprofessional Education and Collaborative Practice Unit at ZUAS) and Dr. Francesco Spöring (Competence Centre for Interprofessional Education, Department of Health Bern, University of Applied Sciences (UAS) Bern. for their support in planning and conducting this meeting at BUAS on February 27, 2020. We would like to thank Dr. David Stamm for his support in translating this manuscript from German into English. Thanks, are also due to the anonymous reviewers whose critical comments helped to improve this manuscript.

## Competing interests

The authors declare that they have no competing interests. 

## Figures and Tables

**Table 1:**
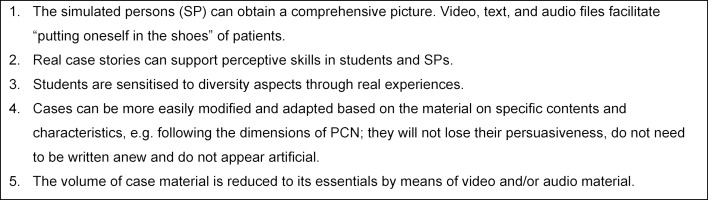
Advantages of embedding real-life DIPEx patient narratives in case development with focus on patient-centredness
